# Hydrogen isotopes reveal evidence of migration of *Miniopterus schreibersii* in Europe

**DOI:** 10.1186/s12898-020-00321-7

**Published:** 2020-09-29

**Authors:** Patrick G. R. Wright, Jason Newton, Paolo Agnelli, Ivana Budinski, Ivy Di Salvo, Carles Flaquer, Antonio Fulco, Panagiotis Georgiakakis, Adriano Martinoli, Maria Mas, Mirna Mazija, Mauro Mucedda, Eleni Papadatou, Boyan Petrov, Luisa Rodrigues, Fiona Mathews, Danilo Russo

**Affiliations:** 1 Vincent Wildlife Trust, Ledbury, HR8 1EP UK; 2 National Environmental Isotope Facility, Scottish Universities Environmental Research Centre, Glasgow, UK; 3 Sistema Museale dell’Università di Firenze, Museo di Storia Naturale, Sede di Zoologia “La Specola”, via Romana 17, 50125 Firenze, Italy; 4 Department of Genetic Research, Institute for Biological Research “Siniša Stanković” - National Institute of Republic of Serbia, University of Belgrade, Bulevar despota Stefana 142, 11060 Belgrade, Serbia; 5 Ecomodel, Rome, Italy; 6 Museu de Ciències Naturals de Granollers, Barcelona, Spain; 7 Dipartimento di Scienze e Tecnologie Biologiche, Chimiche e Farmaceutiche, Laboratorio di Zoologia applicata, Università degli Studi di Palermo, via Archirafi 18, 90123 Palermo, Italy; 8 University of Crete-Voutes Campus, Natural History Museum of Crete, Heraklion, Greece; 9 Unità di Analisi e Gestione delle Risorse Ambientali, Guido Tosi Research Group, Dipartimento di Scienze Teoriche e Applicate, Universita’ degli Studi dell’Insubria,, via J. H. Dunant, 3, 21100 Varese, Italy; 10 Samostalna djelatnost / Freelance Consultant, Koledinečka 3, 10 040 Zagreb, Croatia; 11 Centro Pipistrelli Sardegna, Sassari, Italy; 12 ARUP, 3 Piccadilly Place, Manchester, UK; 13 Divisão de Conservação da Biodiversidade, Instituto da Conservação da Natureza e das Florestas, Lisbon, PT Portugal; 14 University of Sussex, Brighton, BN1 9RH UK; 15 Wildlife Research Unit, Dipartimento di Agraria, Università degli Studi di Napoli Federico II, via Università 100, 80055 Portici (Napoli), Italy

**Keywords:** Chiroptera, Long-distance migration, Stable isotope, Wildlife conservation, Schreiber’s bat, Climate change, Movement ecology

## Abstract

**Background:**

The Schreiber’s bat, *Miniopterus schreibersii*, is adapted to long-distance flight, yet long distance movements have only been recorded sporadically using capture-mark-recapture. In this study, we used the hydrogen isotopic composition of 208 wing and 335 fur specimens from across the species' European range to test the hypothesis that the species migrates over long distances.

**Results:**

After obtaining the hydrogen isotopic composition (δ^2^H) of each sample, we performed geographic assignment tests by comparing the δ^2^H of samples with the δ^2^H of sampling sites. We found that 95 bats out of 325 showed evidence of long-distance movement, based on the analysis of either fur or wing samples. The eastern European part of the species range (Greece, Bulgaria and Serbia) had the highest numbers of bats that had moved. The assignment tests also helped identify possible migratory routes, such as movement between the Alps and the Balkans.

**Conclusions:**

This is the first continental-scale study to provide evidence of migratory behaviour of *M. schreibersii* throughout its European range. The work highlights the need for further investigation of this behaviour to provide appropriate conservation strategies.

## Background

Long-distance migration occurs in several European bat species [1]. Yet the phenomenon is extremely difficult to study as most European bats are too small to carry GPS tags, and capture-mark-recapture approaches are suitable for gathering only incidental records. Understanding migratory patterns is fundamentally important to the assessment of conservation status, and also to the design of appropriate management strategies.

*Miniopterus schreibersii* is included as Near Threatened in the IUCN Red List, and is thought to be migratory at least through some of its range [2]. The species is highly gregarious and philopatric, with both sexes always returning to the roosts in which they were born [1, 3]. Despite the scarce evidence for migration across Europe, in South Africa, the closely related *Miniopterus natalensis* covers up to 560 km to reach its hibernation sites in the north of the country, whereas the southern populations are more sedentary. These behavioural differences are also supported by a higher wing aspect ratio in the migratory individuals [4]. The high aspect ratio of *M. schreibersii* wings suggests that it is adapted to cover long distances [5]. Recorded movements between summer and wintering sites have been highly variable: short distances (e.g. 45 km for both males and females [6, 7]) are recorded, as well as long-distance movements (an individual has been recorded to move 833 km from southern Spain to France (Oficina de Especies Migratorias D.G. de Conservación de la Naturaleza, unpublished data)).

Information to improve the conservation of *M. schreibersii* is urgently needed. Although the species is widely distributed and is common in southern Europe and Asia Minor, it has disappeared from much of the northern part of its range since the 1960s. Recently, unexplained mass mortality events have been observed in south-western Europe, with 40–60% fatality rates being reported in colonies of thousands of individuals [8].

In this study, we used stable hydrogen isotope (δ^2^H) analysis to improve our understanding of *M. schreibersii*’s migratory behaviour. We focused on collecting hair and wing samples (tissues likely to represent different isotopic signatures owing to timing differences in their growth—see “Methods” section) of both male and female bats in spring and autumn throughout the species’ European range. We aimed to (a) identify seasonal, sex and tissue differences in the hydrogen isotopic composition of bats; and (b) infer the geographic origin of all fur and wing samples.

## Results

Season and tissue were both predictors of δ^2^H values (season: X^2^(1) = 37.94, p < 0.001; tissue X^2^(1) = 49.83, p < 0.001) whereas there was no evidence for an effect of sex (X^2^(1) = 1.00, p = 0.316). There was also no evidence that the size of the differences in δ^2^H values between tissues from the same individual differed between either seasons or sexes (season: X^2^(1) = 0.153, p = 0.695; sex: X^2^(1) = 1.49, p = 0.221) (Table 1; Additional file 1: S4, S5 and S6).

**Table 1:** Mean *δ*^2^H values of fur and wing tissue for all 208 *Miniopterus schreibersii* with standard deviations in brackets (see Additional file 1: S1 and S3 for location of sites)

Region	Site	Autumn			Spring		
		*δ*^2^H_*Fur*_	*δ*^2^H_*Wing*_	Δ*δ*^2^H	*δ*^2^H_*Fur*_	*δ*^2^H_*Wing*_	Δ*δ*^2^H
Portugal	1	− 20.37 (3.8)	− 25.04 (4.9)	6.39 (4.3)	− 22.17 (2.6)	− 28.93 (3.2)	6.76 (3.1)
Catalonia	2	− 25.19 (6.9)	− 27.76 (4.4)	4.46 (6.4)	− 24.71 (7.8)	− 25.47 (4.6)	6.97 (4.2)
Italy (Sardinia)	3	− 26.16 (4.6)	− 29.33 (2.3)	4.34 (3.8)	–	–	–
Italy	4	− 39.85 (2.3)	− 33.11 (3.2)	6.74 (2.3)	− 33.72 (2.8)	− 40.35 (6.2)	6.63 (7.1)
Italy	5	− 25.51 (4.3)	− 32.46 (2.9)	6.95 (5.1)	− 30.64 (6.3)	− 31.30 (4.8)	4.75 (4.0)
Italy	6	− 23.14 (1.8)	− 29.51 (5.1)	6.38 (4.2)	− 25.92 (6.3)	− 34.03 (2.1)	8.45 (6.2)
Italy (Sicily)	7	− 18.38 (4.6)	− 23.10 (3.7)	4.85 (5.7)	− 19.93 (5.3)	− 24.53 (4.2)	7.17 (6.0)
Italy	8	− 27.62 (2.5)	− 25.72 (4.7)	2.90 (2.5)	− 31.70 (2.6)	− 30.96 (3.7)	3.76 (2.1)
Croatia	9	− 32.28 (3.5)	− 37.67 (4.1)	5.39 (3.8)	− 43.13 (3.7)	− 43.55 (3.8)	2.61 (1.2)
Serbia	10	− 35.87 (4.8)	− 38.11 (4.2)	5.03 (4.3)	–	–	–
Serbia	11	− 36.07 (3.5)	− 42.25 (5.1)	6.18 (4.3)	− 40.46 (4.8)	− 46.25 (4.3)	7.75 (5.3)
Bulgaria	12	− 38.00 (2.8)	− 39.28 (3.4)	4.08 (2.3)	− 44.46 (3.7)	− 43.60 (4.5)	3.69 (2.5)
Bulgaria	13	− 40.32 (3.4)	− 43.29 (5.1)	5.33 (4.0)	− 41.58 (3.4)	− 43.32 (3.7)	3.03 (3.0)
Bulgaria	14	− 32.92 (4.2)	− 35.71 (3.8)	4.76 (3.7)	− 42.34 (5.9)	− 38.55 (4.7)	5.46 (3.7)
Greece	15	–	–	–	− 17.54 (4.5)	− 29.30 (3.4)	11.77 (5.3)
Greece	16	− 14.8	− 26.9	12.09	− 19.28 (4.7)	− 29.18 (4.5)	9.90 (4.3)

By performing assignment tests for all fur and wing samples, we identified 23.5% (spring: 26.5%; autumn: 20.9%) of wing and 25.1% of fur samples (spring: 27.3%; autumn: 23.0%) as not originating from their sampling site. The combination of the different assignment tests performed resulted in the detection of 95 individual bats classified as ‘non-local’ out of 335.

**Fig. 1:**
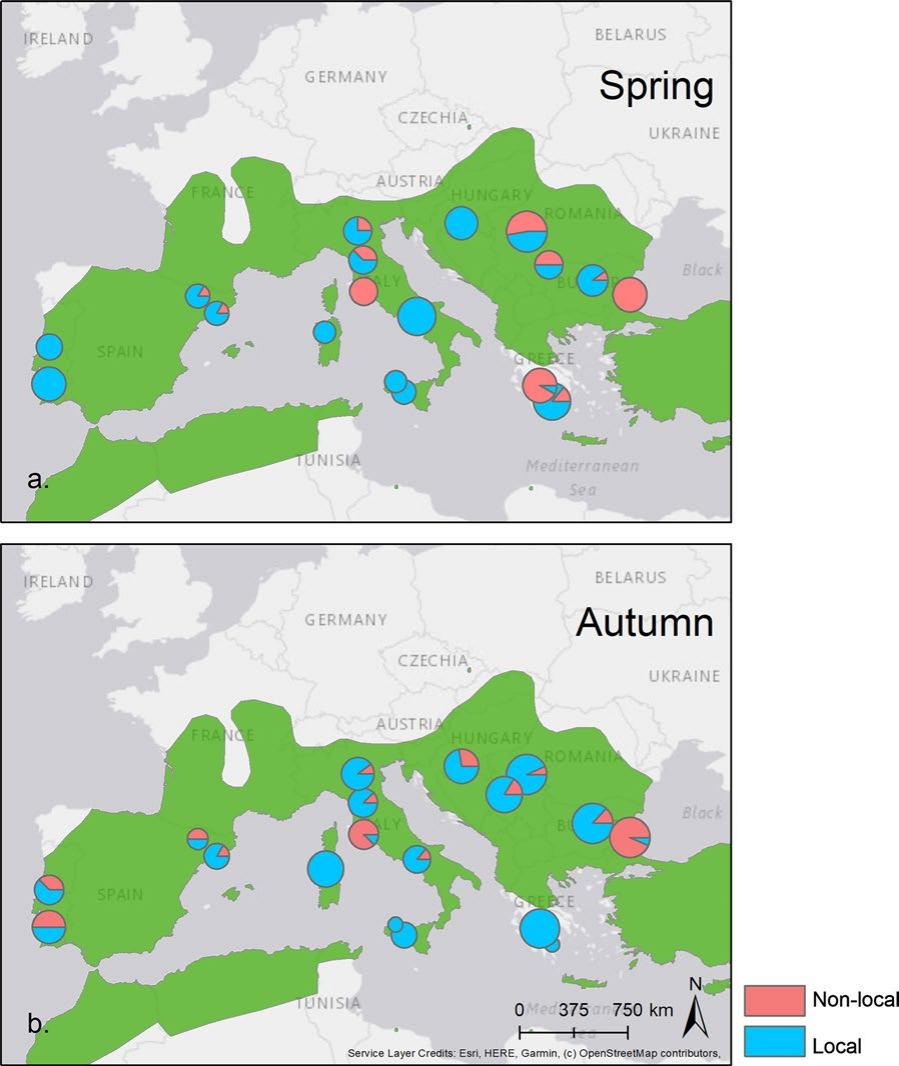
Proportion of *Miniopterus schreibersii* predicted as being ‘local’ and ‘non-local’ at each site in autumn and spring. A bat was classified as ‘non-local’ if either the wing or fur sample was predicted as ‘non-local’. The size of the pie chart is proportional to the sample size. The species’ distribution map as currently described by the IUCN [2] is shown in green

Most bats were predicted as originating from their sampling site throughout both seasons (72%). Only three sites in Tuscany, Greece and Bulgaria were found to have larger numbers of ‘non-local’ bats (Fig. 1). Most sites showed little seasonal variation in the number of ‘non-local’ bats with the exception of the Serbian and Greek sites in spring (a Greek site was not sampled in autumn) and the Portuguese sites in autumn which had a higher number of ‘non-local’ bats (Fig. 1).

**Fig. 2:**
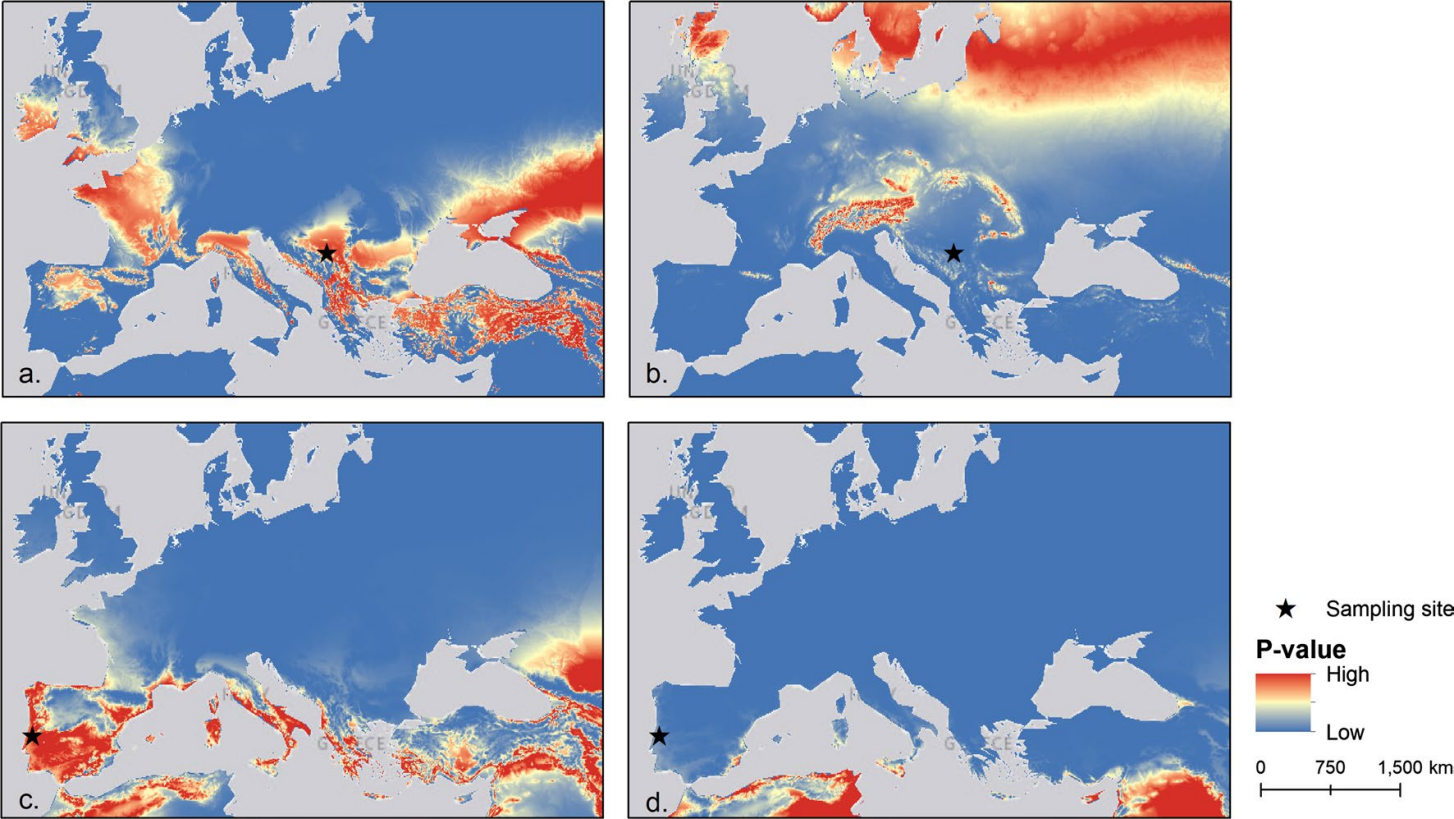
Examples of *Miniopterus. schreibersii* probability (*p* value—values ranging from 0 to 1) maps of geographical assignments (sampling sites in blue areas indicate that the site was rejected as a source of origin for the bat or group of bats). Probability maps of geographical assignment based on **a** a group of bats predicted as being ‘local’; **b** an individual sampled at the same site as **a** predicted as ‘non-local’; **c** an individual bat predicted as being ‘local’ based on the wing sample; and **d** the same individual bat predicted as being ‘non-local’ based on the fur sample

Of the 49 bats identified as ‘non-local’ from the wing samples, very few individuals showed large differences in assignment predictions between fur and wing samples (such as those illustrated in Fig. 2c, d). Indeed, 35 bats out of 49 were also identified as ‘non-local’ when testing fur samples of the same individual (18 in autumn and 17 in spring).

The precise migratory routes for most bats were hard to predict from most sampled sites. However, movement from northern Africa and Portugal (Fig. 2) can be observed from the assignments. Movement from the European Alps was also predicted from multiple sites in eastern Europe and Italy (Fig. 2 and Additional file 1: S10).

## Discussion

Based on the measurement of wing and fur stable hydrogen isotope (δ^2^H), we confirmed that migratory behaviour occurs in the European population of *M. schreibersii*. This is the first continental-scale study performed on the species to provide evidence of long-distance movement throughout its European range. This behaviour appears site-specific—being predominantly found in eastern part of the species' European range—but does not appear to be sex-specific. Our results agree with previous anecdotal records of some bats travelling several hundreds of kilometres (> 800 km) (Oficina de Especies Migratorias D.G. de Conservación de la Naturaleza, unpublished data), but also with the majority of observations of bats undertaking short distance migration between summer and winter roosts (~ 50 km; [6]).

Most migratory European bats travel in a northeast-southwest direction [9]. Latitudinal migrations are better detected than longitudinal migrations when analysing stable hydrogen isotope data [10, 11]. Therefore, the assignment of a Mediterranean species showing east/west range, such as *M. schreibersii*, is often ambiguous. For example, migratory routes in Greece were hard to characterise, as high probabilities of assignment were identified in Greece, but also in Portugal and northern Africa, providing multiple alternative routes (Additional file 1: S9). Nonetheless, our findings allow us to identify some possible migratory routes for *M. schreibersii*. Many of the migrant bats from Serbia and Bulgaria showed high probabilities of origin in northern Europe. The absence of the species from most of the latter region suggests that bats are likely to originate from the Alps, an area where *M. schreibersii* is known to be present. Movement from southern Spain to France (> 800 km) has been confirmed for a single bat in the past by using banding data (Oficina de Especies Migratorias D.G. de Conservación de la Naturaleza, unpublished data). Our results suggest that this could be a migratory route as multiple bats from Catalonia were shown to be likely to originate from southern Portugal and northern Africa. Other long-distance migrations inferred from our results suggested possible movement from mainland Italy to Sardinia; and movement between southern Europe and northern Africa. The ability to detect movements from northern Africa may be particularly relevant when predicting future range-shifts of the species in response to climate change, and highlight the ability of these bats to cross the Mediterranean—something previously not possible to demonstrate owing to the very small number of banding studies being conducted.

The collection of samples from two tissue types with different turnover rates had the potential to reflect the isotopic signature of an individual at different points in time. While most individual bats showed little differences in fur and wing geographic assignments, these results could vary considerably at certain sites. For a small number of individuals, δ^2^H_fur_ had a stronger tie with their sampling site than did δ^2^H_wing_. The fact that wing samples are likely to reflect a more recent isotopic signature than fur samples (see “Methods” section) indicates that these individuals had recently returned to the sites where their fur had developed (likely to be June–August) after travelling significant distances. Such behaviour could be driven by the need to visit multiple sites for mating [12], to assess the condition of hibernacula, and/or transfer information to juveniles on the location of traditional roosting sites [13].

In comparison to birds, the migratory behaviour of most bat species remains largely unknown. However, differential migration, where populations migrate separately (sex-, age- or other subgroups), as opposed to random mixing, is thought to be the dominant pattern. Our results suggest regional differences but no obvious sex differences. Yet, *M. schreibersii* short-distance migration timing is known to vary according to sex and age [6]. In Greece, where mostly migratory males were sampled, the absence of females may be a result of differing migration timings as the latter are also known to use these sites.

The creation of geographical assignments from stable isotopes has limitations in terms of geographical resolution of baseline isoscape data, poorly constrained transfer functions (i.e. the relationship between rainwater δ^2^H and tissue δ^2^H), lack of information on the dietary intakes of different stable isotopes owing to evidence gaps for foraging behaviour of volant species [14], and the relative lack of variability in water isoscapes over large regions. Hence, only major advances with the miniaturisation of GPS-tag technologies and/or the creation of arrays of static receiver stations for VHS radio-tags will provide a better understanding of *M. schreibersii* migratory behaviour.

Roost temperature is a key driver explaining short-distance migration for *M. schreibersii* [6]. As climate change affects the distribution and survival of many bat species [15], more bats may undertake long-distance migration to find suitable roosts. The overall cost of long-distance migration—increased energy expenditure, greater exposure to anthropogenic threats [16, 17] and increased risk for the spread of diseases [18]—could substantially impact populations. Therefore, a better understanding of migratory routes and the drivers behind long-distance migration is essential.

## Conclusions

Our results show that an important number of *M. schreibersii* bats undertake long-distance migration. This behaviour is observed through the species’ European range, but it also appears to be more common at some sites. This information is a first essential step towards better protecting this species, and demonstrates the utility of stable isotopes in informing landscape-scale conservation of bats more widely.

## Methods

### Sample collection

Bats were captured near roosts by mist nets or harp traps depending on roost characteristics, colony size and other local features. We collected 335 fur samples of *M. schreibersii* from 20 sites across southern Europe during spring and autumn 2015 (Additional file 1: S1). Wing biopsy punches were also taken from 212 of the 335 individuals sampled and stored in ethanol. All samples were collected under the appropriate licence of each country. After establishing sex, age class and taking other biometric measurements, bats were released at their capture location.

### Stable isotope analysis

The same protocols were applied for wing and fur samples. Prior to analysis, we rinsed samples in 2:1 chloroform/methanol solution for 24 h, and repeated this a second time for 1 h. We then rinsed samples in ultra-pure water to remove all solvents and oils before letting them to dry at 44 °C (Additional file 1: S2). Samples were added to 5 × 3.5 mm silver capsules and were weighed to 0.15 mg (±0.05 mg) or less if insufficient material had been collected.

We loaded encapsulated samples and standards into a Eurovector UniPrep autosampler [19] and pumped at 60 °C for two 1-h periods separated by a 10-min break in a helium atmosphere. This ensures the removal of residual adsorbed moisture. Hydrogen in the samples was converted to H_2_ gas in the reactor of a Thermo Fisher Scientific TC/EA—a high-temperature thermal conversion elemental analyser (HTC-EA). The reactor was filled with chromium powder and glassy carbon (following Gehre, Hoefling [20]), which prevents the formation of other hydrogenous gases such as hydrogen cyanide (HCN), a likely product of the thermal conversion of keratin. Then, δ^2^H was measured on the resulting H_2_ on a Thermo Fisher Scientific Delta V Plus isotope ratio mass spectrometer.

Since a proportion of keratin contains H, which is exchangeable with ambient water vapour, we compared samples to matrix-equivalent reference materials which have known non-exchangeable δ^2^H to determine the non-exchangeable δ^2^H of the samples (no bat wing membrane standards were available for this study). The reference materials used were USGS42 and USGS43 hair (− 72.2 ± 0.9 and − 44.2 ± 1.0‰ respectively Soto, Koehler [21]. In addition, ground Maltese goat hair samples of unknown δ^2^H were added to each run as an independent assessment of quality control over the 4-week period of analysis. Repeated samples of the goat hair (n = 39) amongst the sample δ^2^H measurements gave a standard deviation of 1.65‰ (n = 39). All standards were analysed in triplicate in each run.

### Statistical analysis and regional assignments

We undertook all statistical analyses in the R software (v. 3.4.3; [22]) and implemented them in R Studio (v. 3.5.1; [23]). We used lme4 [24] to perform a linear mixed effects analysis of the relationships between δ^2^H values and tissue type, season and sex (fixed effects) from individuals for which both tissue types were collected (n = 208). Site and individual ID were included as random effects. Then, we assessed the relationship between the difference in δ^2^H values between tissues from the same individual (Δ*δ*^2^H = |δ^2^H_wing _− δ^2^H_fur_|) with season and sex using site and individual ID as random effects. We obtained p-values by likelihood ratio tests of the full model against the models without the tested effects.

Isoscapes represent the spatial patterns of stable isotope ratios and helps with the interpretation and visualisation of data [25, 26]. Here, we made geographic assignments using the R package *IsoriX* which constructs isoscapes and assigns the origin of organisms based on their isotopic signature [27, 28]. Measurements of rainfall δ^2^H ranging from 2005 to 2017 during the months of June, July and August from the GNIP database (https://websso.iaea.org/) were used to create a spatial mixed model predicting isoscape. As bats in Europe and America tend to moult between June and August, origin assignments of fur samples should be representative of the preceding summer [29, 30]. Little is known about the timing and growth of wing membranes, but it is likely to be dependent on the bat’s metabolism during the preceding months or weeks (i.e. hibernating, breeding period). However, as a fast healing tissue which regenerates in 2−3 weeks during the active season, they are likely to reflect a more recent isotopic signature than fur samples [31, 32].

We performed assignment tests for all fur and wing isotopic values, using comparisons against the summer isoscape (Additional file 1: S7 and S8). In the absence of data from a sedentary species that could be used for the calibration fit (transfer function—the relationship between sedentary bats δ^2^H against precipitation at those sites), we used data from individuals showing differences between wing and fur δ^2^H inferior to the standard deviation of the goat hair standard (< 1.65‰) as these were assumed to be sedentary animals. Then, we applied the transfer function (δ^2^H_fur_ = 0.62 δ^2^H_isoscape_ − 14.66; δ^2^H_wing_ = 0.64 δ^2^H_isoscape_ − 14.64, Additional file 1: S9) between the sample δ^2^H values and rainfall isoscape δ^2^H values obtained from the sedentary animals using the ‘Calibfit’ function to assign the origin of both fur and wing samples. Here, we classified a bat as ‘non-local’ if either the wing or fur sample was predicted as ‘non-local’.

## Supplementary information

**Additional file 1.** Summary of sampling sites and result outputs.

## Data Availability

All data are available on the Figshare digital repository (DOI: 10.6084/m9.figshare.12369191.v1).
