# Supplementary material for: Hydrogen isotopes reveal evidence of migration of Miniopterus schreibersii in Europe
Source: BMC Ecol. 2020 Sep 29;20:52. doi: 10.1186/s12898-020-00321-7 (PMC7526252; doi:10.1186/s12898-020-00321-7)

Additional information – Summary of sites and result outputs

**Hydrogen isotopes reveal evidence of migration of *Miniopterus schreibersii* in Europe**

Patrick G. R. Wright, Jason Newton, Paolo Agnelli, Ivana Budinski, Ivy Di Salvo, Carles Flaquer, Antonio Fulco, Panagiotis Georgiakakis, Adriano Martinoli, Maria Mas, Mirna Mazija, Mauro Mucedda, Eleni Papadatou, Boyan Petrov, Luisa Rodrigues^14^, Fiona Mathews* & Danilo Russo

Contents

[Additional file 1: S1 - Distribution map of *M. schreibersii* as currently described by the IUCN with location of sampled sites. 2](#_Toc50909528)

[Additional file 1: S2 - Sample preparation protocol 2](#_Toc50909529)

[Additional file 1: S3 - List of sampling sites and coordinates. 3](#_Toc50909530)

[Additional file 1: S4 - Table showing mean δ^2^H values of all fur tissues from each site 4](#_Toc50909531)

[Additional file 1: S5 - Table summarising the results from the mixed effects analyses. 4](#_Toc50909532)

[Additional file 1: S6 - Box plot showing differences in δ^2^H between sex and season for both wing and fur samples. 5](#_Toc50909533)

[Additional file 1: S7 - Relationship between the observed and predicted response corresponding to the fit of the mean of isotopic values for the summer rainfall δ^2^H isoscape. 5](#_Toc50909534)

[Additional file 1: S8 - Prediction of the average isotope value (mean δ^2^H) and the temporal variation at each location (mean residVar δ^2^H) for the summer rainfall δ^2^H isoscape. 6](#_Toc50909535)

[Additional file 1: S9 - Table summarising results from the calibration data used for the assignment of individual bats where the slope results from fixed effect estimates of the calibration fit. The sedentary individuals were determined by the difference between wing and fur δ^2^H, if the difference was < 1.65 ‰ then the individual was assumed to be sedentary (See methods). 6](#_Toc50909536)

[Additional file 1: S10 - Assignment plots of each bat identified as not originating from the site they were sampled from. Individuals are classified in categories associated with tissue type and season (e.g. Fur – Autumn = Fur samples collected in autumn). The title of each map represents the individual ID and the country of origin of the sample. The first map below, for example, represents the assignment plot of a fur sample collected from bat ALV102 in Autumn and in Portugal. 7](#_Toc50909537)


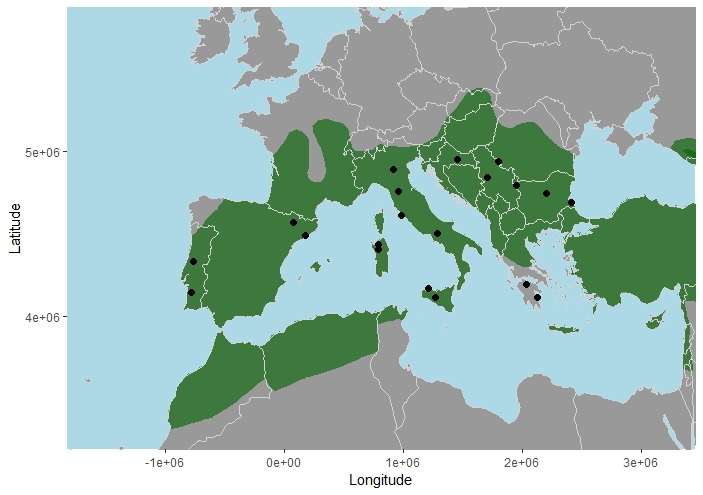


## Additional file 1: S1 - Distribution map of *M. schreibersii* as currently described by the IUCN with location of sampled sites.

## Additional file 1: S2 - Sample preparation protocol

- soak hair in 1ml of 2:1 chloroform:methanol solution for 24 hours in a 1.5ml tube

- remove the solvent and add 1ml of fresh 2:1 chloroform:methanol to each tube

- shake it well to rinse

- remove solvent

- add 400ul of ultra-pure water

- shake well to rinse

- remove water

- let it fully dry out at 44°C

**Very important:** make sure all samples are treated exactly the same way, if possible with same batch of solvents and to rinse thoroughly with distilled water.

## Additional file 1: S3 - List of sampling sites and coordinates.

| **Region** | **Site** | **Lat** | **Lon** |
| --- | --- | --- | --- |
| Portugal | 1 | 39.83198 | -8.41042 |
| Portugal | 1a | 38.03146 | -8.42327 |
| Catalonia | 2 | 41.42644 | 2.021928 |
| Catalonia | 2a | 42.22429 | 0.846 |
| Italy (Sardinia) | 3 | 40.81789 | 8.80931 |
| Italy (Sardinia) | 3a | 40.5215 | 8.74769 |
| Italy | 4 | 45.16081 | 10.8 |
| Italy | 5 | 43.8694 | 11.13521 |
| Italy | 6 | 42.43249 | 11.17225 |
| Italy (Sicily) | 7 | 37.63794 | 13.68185 |
| Italy (Sicily) | 7a | 38.15092 | 13.1559 |
| Italy | 8 | 41.2795 | 14.49113 |
| Croatia | 9 | 45.49594 | 17.26319 |
| Serbia | 10 | 45.12826 | 21.32164 |
| Serbia | 11 | 44.24517 | 19.93554 |
| Bulgaria | 12 | 43.65178 | 22.70244 |
| Bulgaria | 13 | 42.94778 | 25.43037 |
| Bulgaria | 14 | 42.28736 | 27.74987 |
| Greece | 15 | 37.959 | 22.1402 |
| Greece | 16 | 37.1662 | 22.8988 |

## Additional file 1: S4 - Table showing mean δ^2^H values of all fur tissues from each site

|  |  | **Spring** | | **Autumn** | |
| --- | --- | --- | --- | --- | --- |
| **Region** | **Site** | **δ^2^H** | **n** | **δ^2^H** | **n** |
| Portugal | 1a | - | - | -13.00 (3.2) | 9 |
| Portugal | 1 | -22.17 (2.6) | 7 | -20.37 (3.8) | 8 |
| Catalonia | 2 | -33.77 (5.6) | 6 | -29.86 (5.7) | 4 |
| Catalonia | 2a | -24.71 (7.8) | 6 | -25.19 (6.9) | 6 |
| Italy (Sardinia) | 3a | -29.19 (2.3) | 5 | -24.62 (3.1) | 5 |
| Italy (Sardinia) | 3 | - | - | -25.92 (5.3) | 12 |
| Italy | 4 | -37.06 (5.6) | 8 | -39.38 (3.4) | 9 |
| Italy | 5 | -33.78 (6.4) | 7 | -28.15 (4.5) | 7 |
| Italy | 6 | -26.03 (5.1) | 8 | -24.71 (3.4) | 7 |
| Italy | 8 | -32.29 (2.6) | 15 | -29.62 (6.1) | 7 |
| Italy (Sicily) | 7a | -21.16 (2.7) | 5 | -19.54 (2.4) | 5 |
| Italy (Sicily) | 7 | -19.93 (5.3) | 5 | -18.38 (4.6) | 6 |
| Croatia | 9 | -43.39 (3.7) | 10 | -33.83 (7.2) | 10 |
| Serbia | 10 | - | - | -35.68 (4.6) | 12 |
| Serbia | 11 | -44.09 (5.0) | 17 | -38.95 (3.4) | 15 |
| Bulgaria | 12 | -42.81 (5.5) | 12 | -32.92 (4.2) | 14 |
| Bulgaria | 13 | -43.67 (3.4) | 11 | -38.11 (2.8) | 14 |
| Bulgaria | 14 | -41.58 (3.4) | 9 | -40.21 (3.3) | 14 |
| Greece | 15 | -17.54 (4.5) | 12 | - | - |
| Greece | 16 | -21.88 (5.8) | 14 | -14.78 | 1 |

## Additional file 1: S5 - Table summarising the results from the mixed effects analyses.

|  | AIC | X^2^ | p-value |
| --- | --- | --- | --- |
| δ^2^H ~ Tissue + Sex + Season+ (1\|Site) + (1\| ID) | 2543.7 | - | - |
| Null Season | 2579.6 | 37.94 | <0.001 |
| Null Tissue | 2591.5 | 49.83 | <0.001 |
| Null Sex | 2542.7 | 1 | 0.316 |
| ∆δ^2^H_tissue_ ~ Sex + Season+ (1\|Site) + (1\| ID) | 1354.8 | - | - |
| Null Sex | 1354.3 | 1.49 | 0.221 |
| Null Season | 1353 | 0.153 | 0.695 |


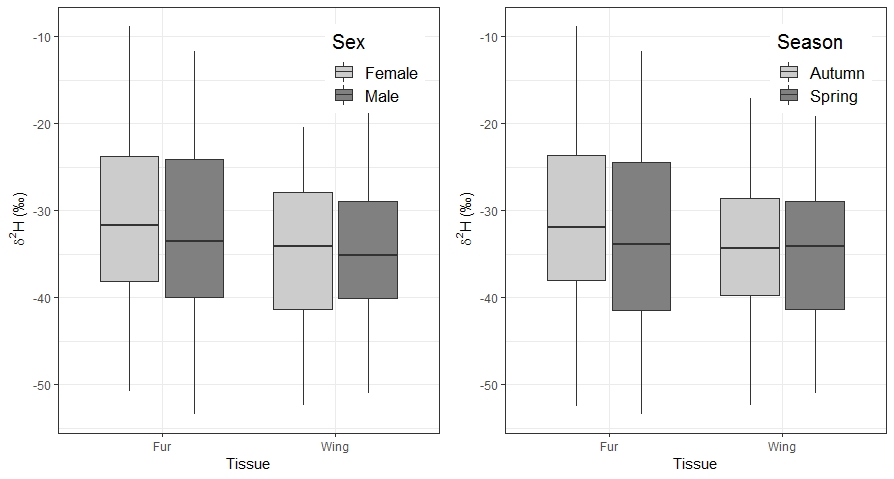


## Additional file 1: S6 - Box plot showing differences in δ^2^H between sex and season for both wing and fur samples.


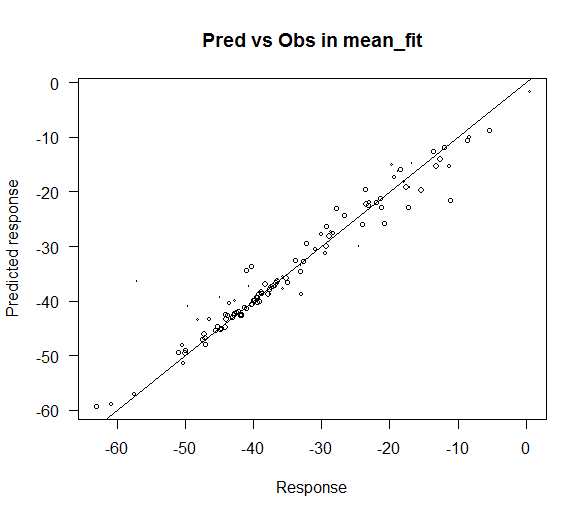


## Additional file 1: S7 - Relationship between the observed and predicted response corresponding to the fit of the mean of isotopic values for the summer rainfall δ^2^H isoscape.


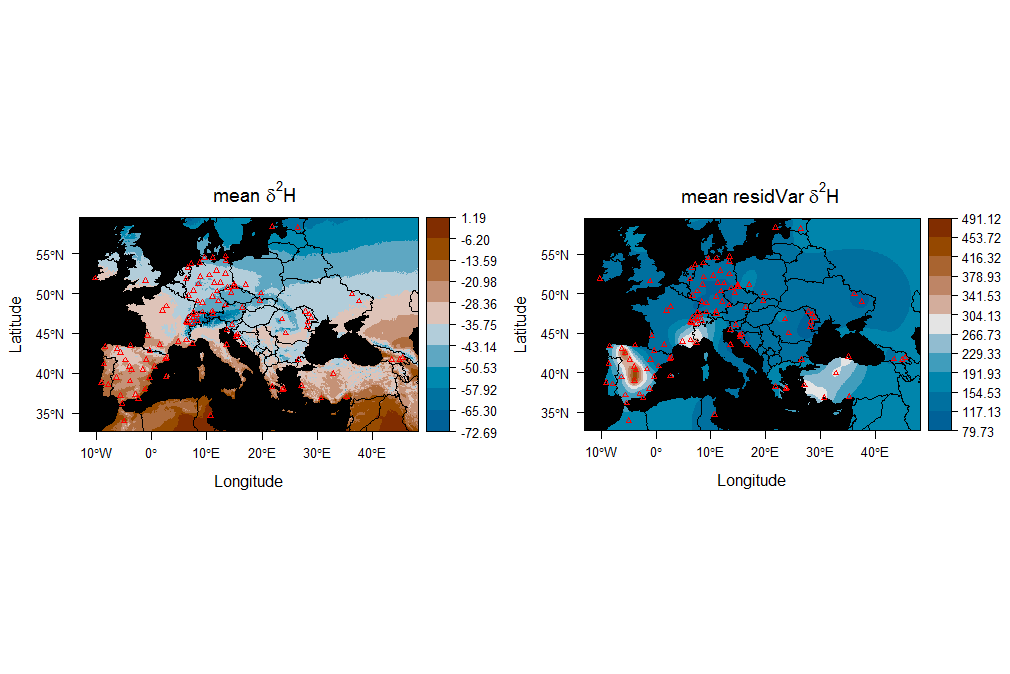


## Additional file 1: S8 - Prediction of the average isotope value (mean δ^2^H) and the temporal variation at each location (mean residVar δ^2^H) for the summer rainfall δ^2^H isoscape.

## Additional file 1: S9 - Table summarising results from the calibration data used for the assignment of individual bats where the slope results from fixed effect estimates of the calibration fit. The sedentary individuals were determined by the difference between wing and fur δ^2^H, if the difference was < 1.65 ‰ then the individual was assumed to be sedentary (See methods).

| **Samples** | **Tissue** | **Slope** | **Intercept** |
| --- | --- | --- | --- |
| Sedentary individuals | Fur | 0.62 +/- 0.10 | -14.66 +/- 3.11 |
| Sedentary individuals | Wing | 0.64 +/- 0.10 | -14.64 +/- 3.15 |
| All samples | Fur | 0.78 +/- 0.13 | -15.82 +/- 2.87 |
| All samples | Wing | 0.64 +/- 0.10 | -8.20 +/- 3.72 |

## Additional file 1: S10 - Assignment plots of each bat identified as not originating from the site they were sampled from. Individuals are classified in categories associated with tissue type and season (e.g. Fur – Autumn = Fur samples collected in autumn). The title of each map represents the individual ID and the country of origin of the sample. The first map below, for example, represents the assignment plot of a fur sample collected from bat ALV102 in Autumn and in Portugal.

Fur – Autumn


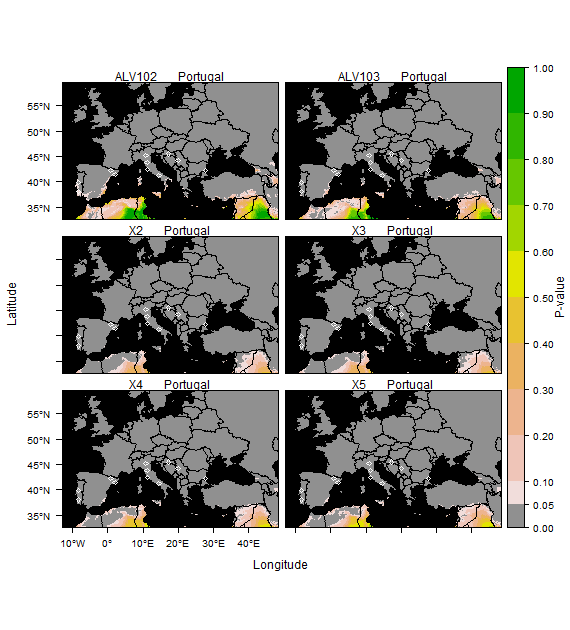


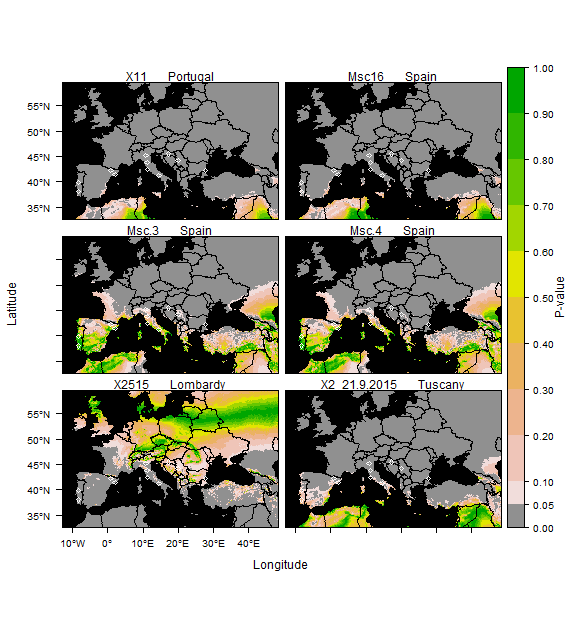

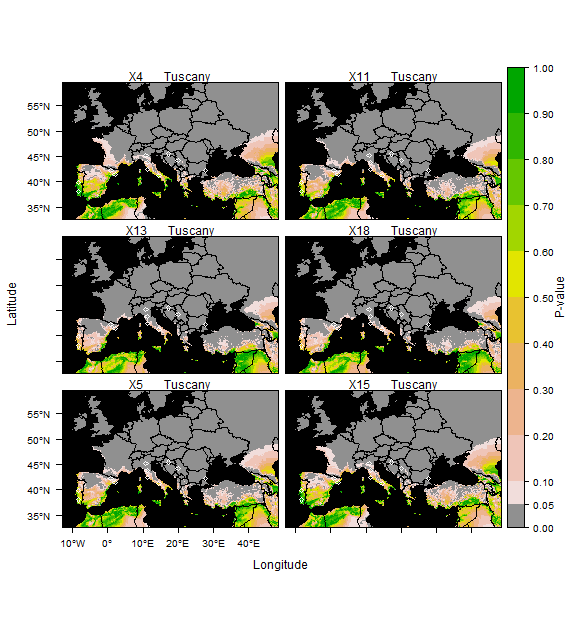


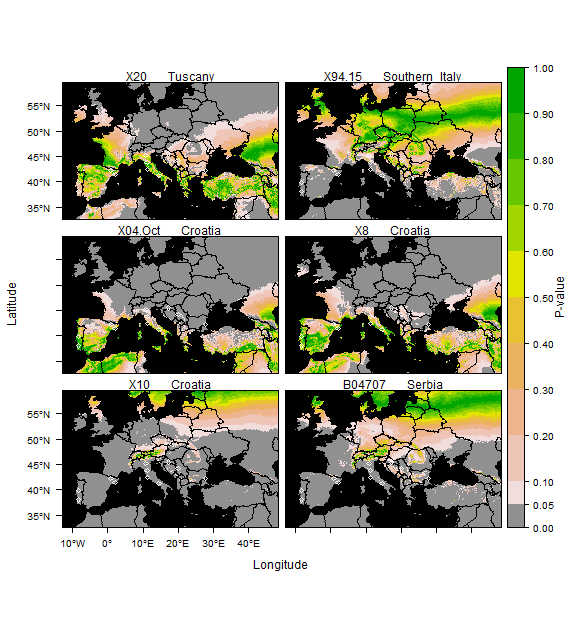


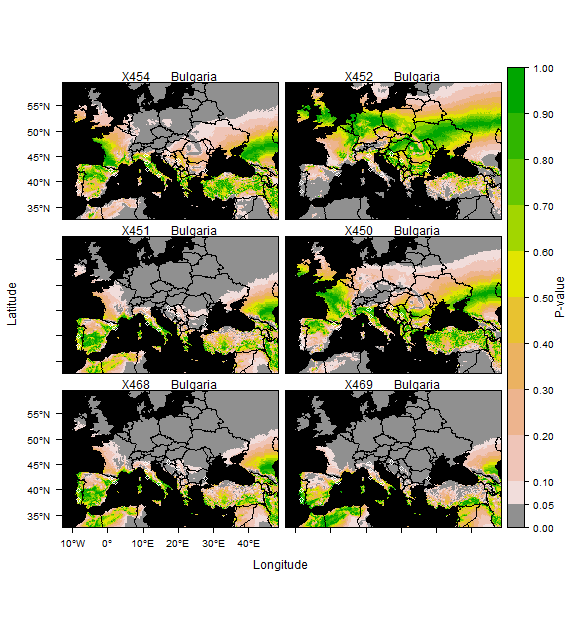


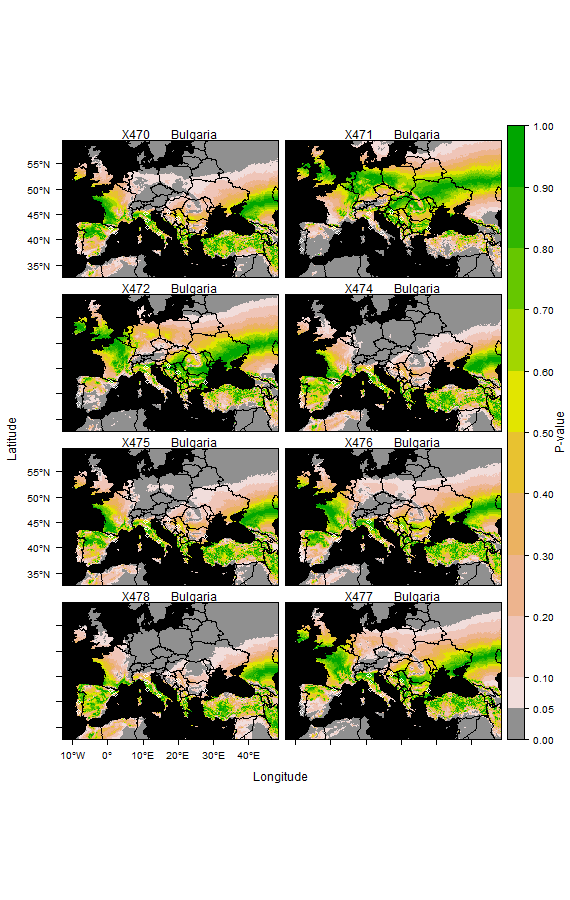


Fur-Spring


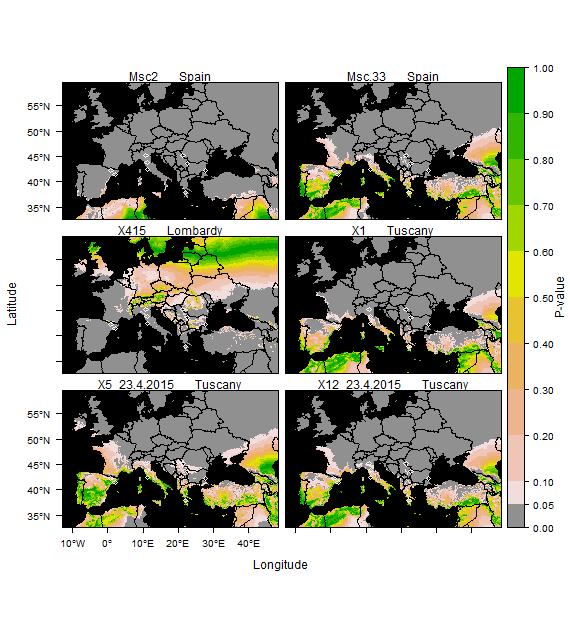


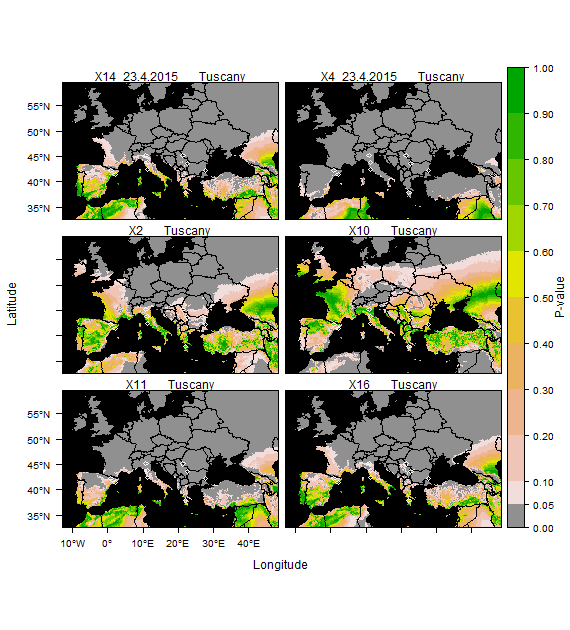


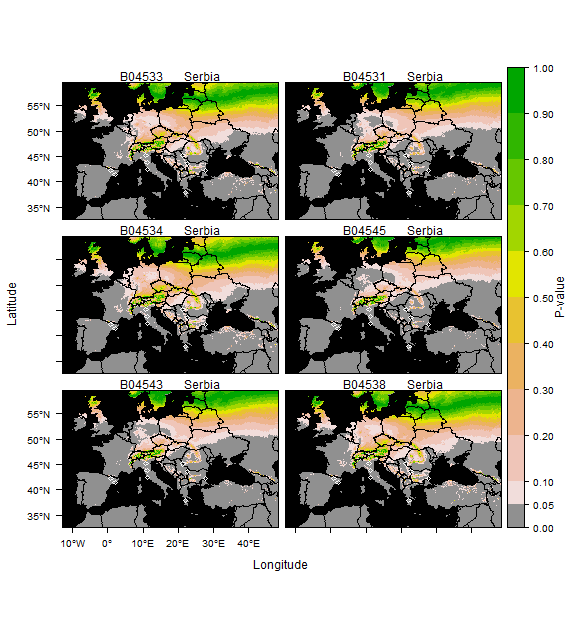


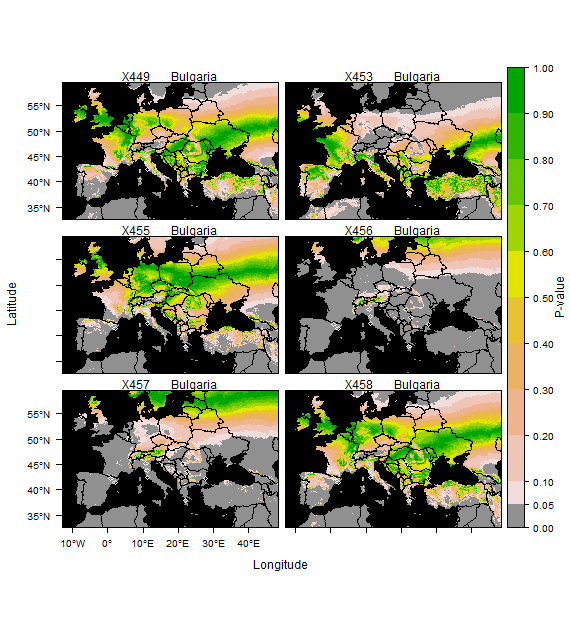


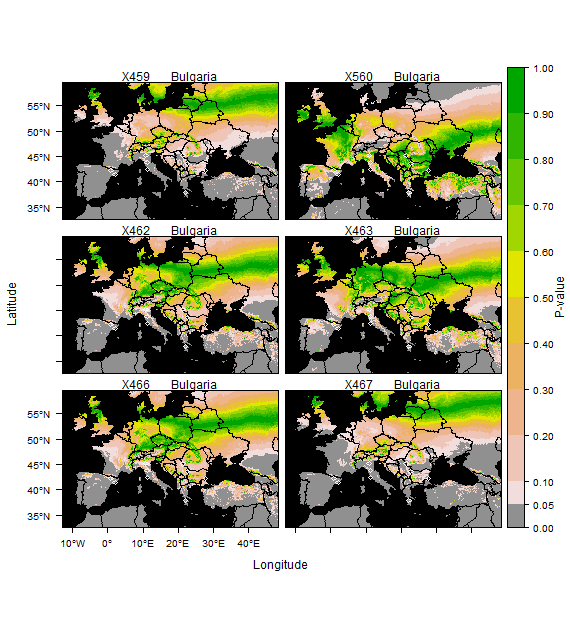


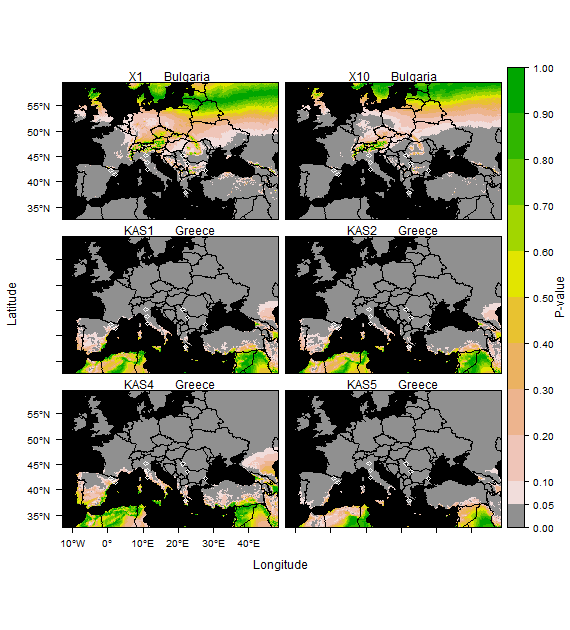


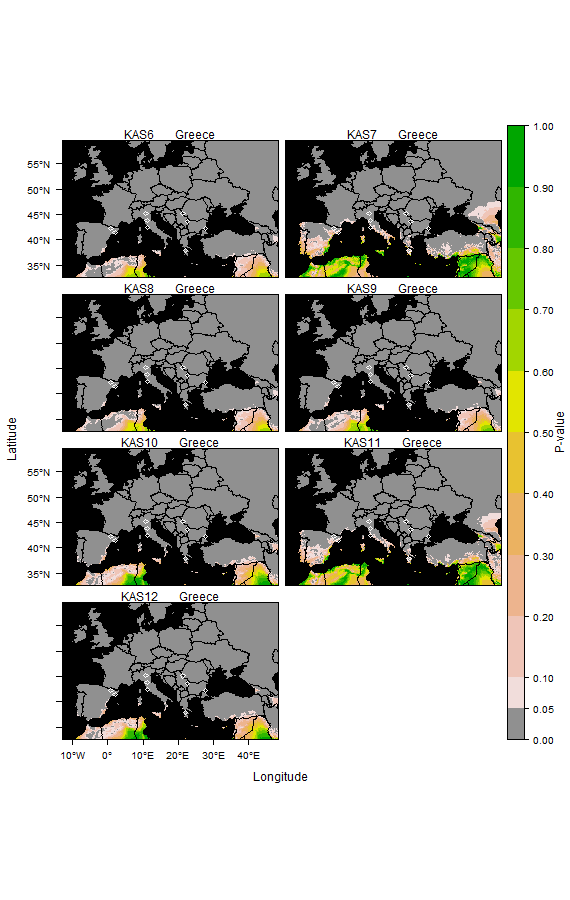


Wing-Autumn


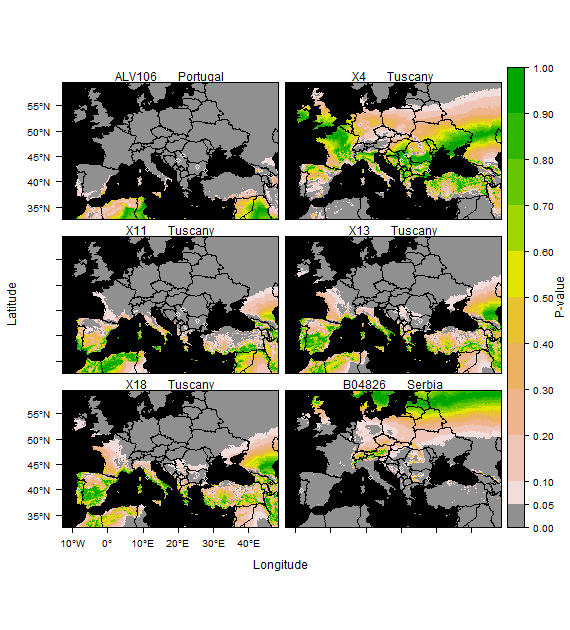


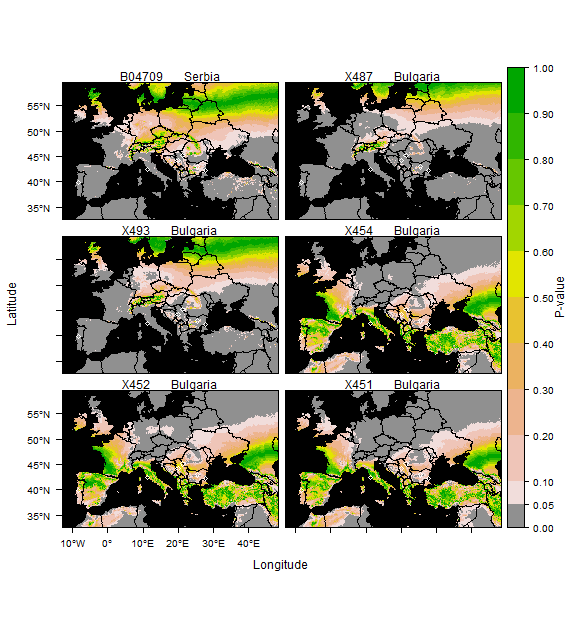


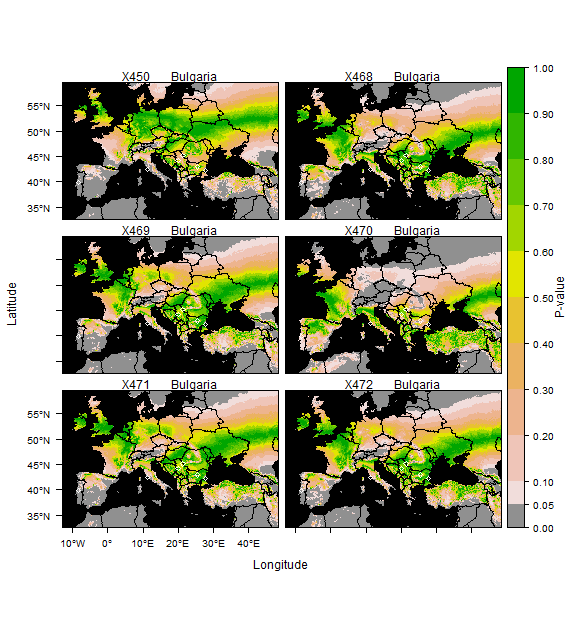

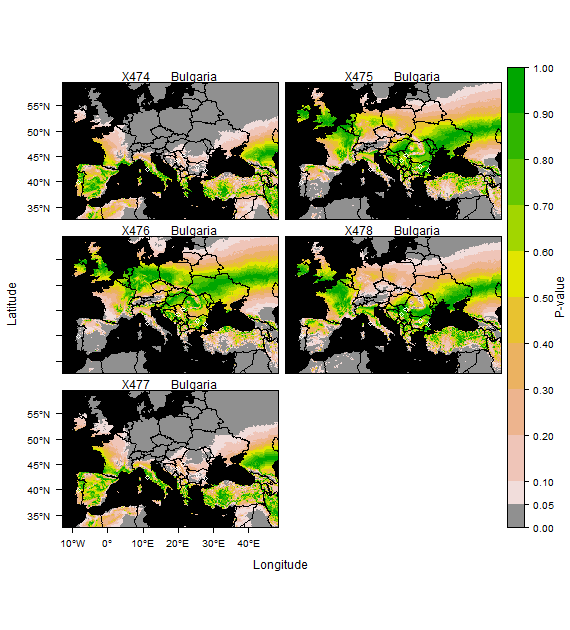


Wing-Spring


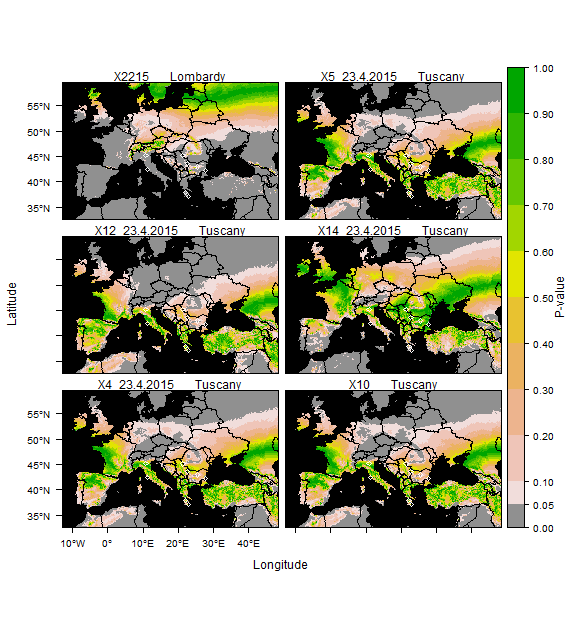


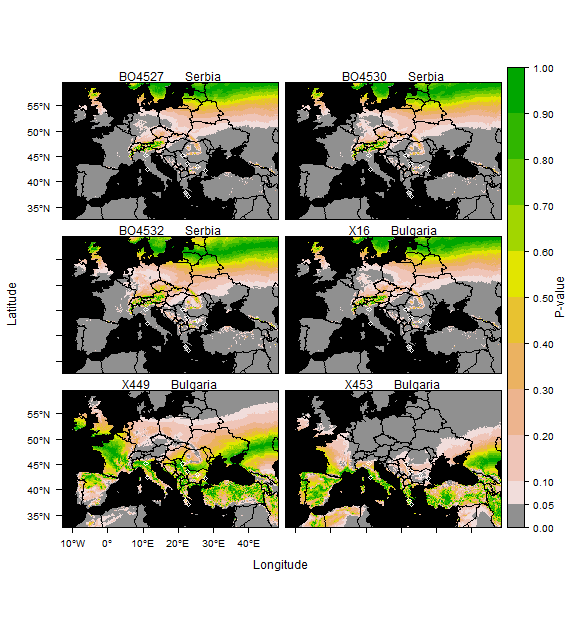

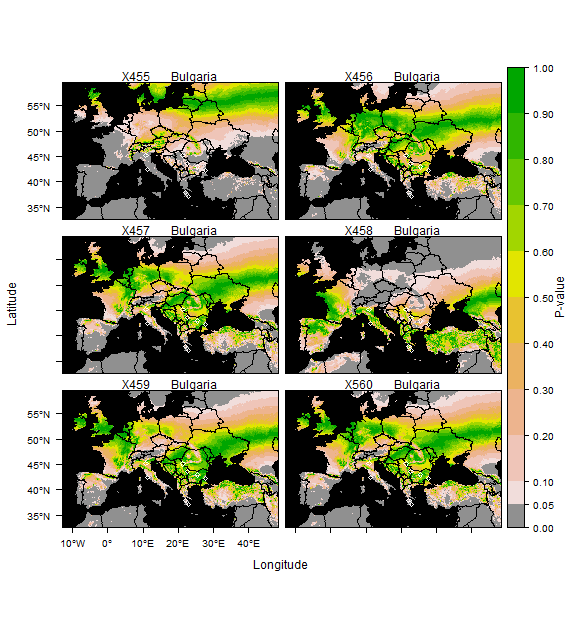

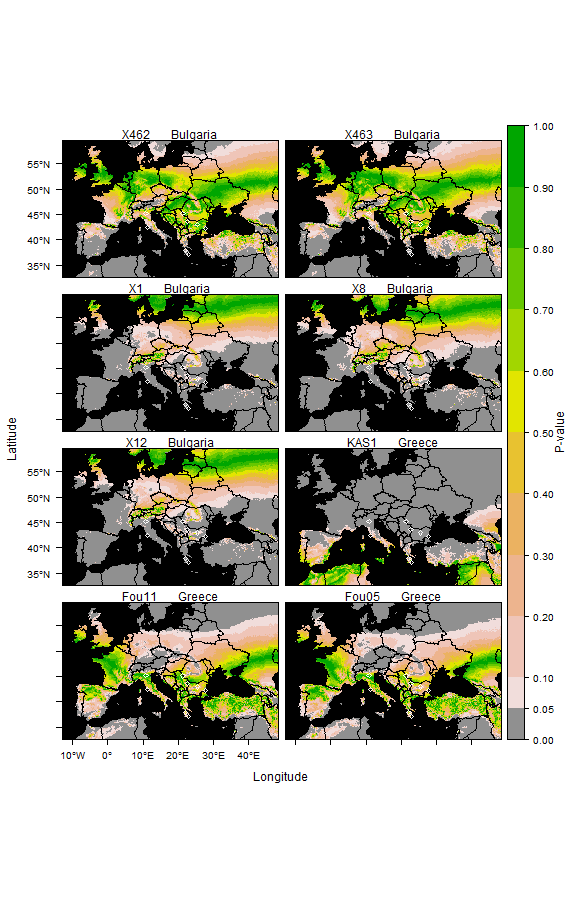

Supplement: Supplementary file 1 — Additional file 1. Summary of sampling sites and result outputs. [file 12898_2020_321_MOESM1_ESM.docx]
